# Dendritic cells in atherosclerosis: integrating ontogeny, functional states, spatial organization, and therapeutic implications

**DOI:** 10.3389/fimmu.2026.1968525

**Published:** 2026-10-20

**Authors:** Wei Shao, Yunxian Zhang, Huasong Luo, Jinbai Huang

**Affiliations:** 1 Yangtze University, First Affiliated Hospital of Yangtze University, Jingzhou, China; 2 Yangtze University Health Science Center, Yangtze University, Jingzhou, China

**Keywords:** atherosclerosis, dendritic cells, single-cell transcriptomics, spatial immunology, therapeutic modulation

## Abstract

Atherosclerosis arises from the interaction between lipid accumulation and sustained innate and adaptive immune activation, and residual cardiovascular risk despite intensive lipid-lowering therapy underscores the need to clarify vascular inflammatory mechanisms. Dendritic cells (DCs) link antigen sensing and presentation to T-cell regulation, but their roles in atherosclerosis remain incompletely resolved. This review synthesizes evidence on conventional type 1 and type 2 DCs, plasmacytoid DCs, and monocyte-derived DC-like cells using a lineage–state–location–validation framework that asks which cells are present, which functional programs they exhibit, where they are located, and what evidence supports the proposed function. Current evidence indicates that lineage defines functional potential but does not determine pathological activity; lipid loading, oxidative and metabolic stress, extracellular nucleic acids, cytokines, anatomical compartment, and disease stage can shift DCs toward inflammatory, regulatory, or dysfunctional states. Single-cell and spatial profiling have improved resolution of plaque-associated myeloid populations, but rare human DCs, overlap with macrophages and monocytes, cross-sectional sampling, and the inability of expression or proximity to establish causality remain important limitations. Therapeutic strategies should be distinguished as direct or DC-enriched delivery, state- or pathway-directed modulation, and indirect effects on DC function. Current interventions remain preclinical and generally lack sufficient cellular or state selectivity. Future work should prioritize reproducible human DC-state identification, lineage-resolved spatial and functional validation, established-disease models, and biomarkers of pathway activity and target engagement. No specific human DC state is yet clinically actionable.

## 1 Introduction

Atherosclerotic cardiovascular disease remains a leading cause of mortality worldwide (1). Intensive low-density lipoprotein cholesterol lowering reduces major vascular events, yet substantial residual cardiovascular risk persists (2, 3). Persistent inflammatory mechanisms contribute to plaque progression and recurrent events even when lipid-related risk is treated (4–7).

Atherosclerotic lesions contain multiple T-cell subsets with distinct inflammatory or regulatory functions, including proinflammatory T helper 1 (Th1) and T helper 17 (Th17) cells and regulatory T cells (Tregs) (7–10). CD8^+^ T cells and other lymphocyte populations have also been implicated in vascular injury and plaque inflammation (11–14). How these responses are initiated, polarized, maintained, and spatially organized within the diseased arterial wall remains an important unresolved question (15, 16).

Dendritic cells (DCs) are positioned upstream of these processes (17, 18). As professional antigen-presenting cells, they coordinate antigen presentation, costimulation, and cytokine signaling to regulate T-cell activation and differentiation, thereby linking innate immune sensing to adaptive immunity (19–21). Within atherosclerotic lesions, DC function is further shaped by lipid uptake and interactions with vascular and immune cells (22, 23). Their effects are context dependent: some DC populations promote inflammatory T-cell responses, whereas others support immune regulation. Experimental studies of conventional type 1 dendritic cells (cDC1s) and plasmacytoid dendritic cells (pDCs) have produced proatherogenic, neutral, or protective outcomes depending on the depletion strategy, disease model, and immune pathway examined (24–28). These findings indicate that lineage identity alone does not fully predict DC activity in atherosclerosis.

Conditional knockout models, single-cell transcriptomics, and spatially resolved analyses have provided a more nuanced view of DC heterogeneity (29). These approaches indicate that lineage identity defines functional potential but does not fully determine pathological activity. Cells within a related DC population may acquire distinct programs in response to metabolic, inflammatory, and tissue-derived signals. Atherosclerotic plaques are also spatially heterogeneous, as shown by human single-cell and spatial analyses (30, 31). Intimal plaque regions and adventitial artery tertiary lymphoid organs (ATLOs) constitute different cellular environments that expose antigen-presenting cells to distinct combinations of antigens, metabolites, cytokines, and interacting cells (32, 33). DC activity should therefore be interpreted according to lineage identity, functional state, and anatomical context rather than inferred from surface phenotype alone.

Previous reviews have summarized DC classification and function in atherosclerosis, DC–macrophage overlap, the context-dependent roles of pDCs, and the broader immune landscape of atherosclerotic plaques (34). More recent reviews have emphasized insights derived from single-cell and spatial technologies (35, 36). Building on this work, we apply a lineage–state–location–validation framework organized around four questions: what is the developmental identity of the cell, which functional program does it exhibit, where is it located, and what evidence supports the proposed function? We use these questions to compare divergent experimental findings and human datasets, distinguish established mechanisms from candidate states and spatial hypotheses, and assess whether therapeutic effects reflect direct DC targeting or indirect modulation. The framework provides a structure for evaluating evidence rather than a validated classification of plaque DCs. **Figure 1** summarizes this approach.

**Figure 1:**
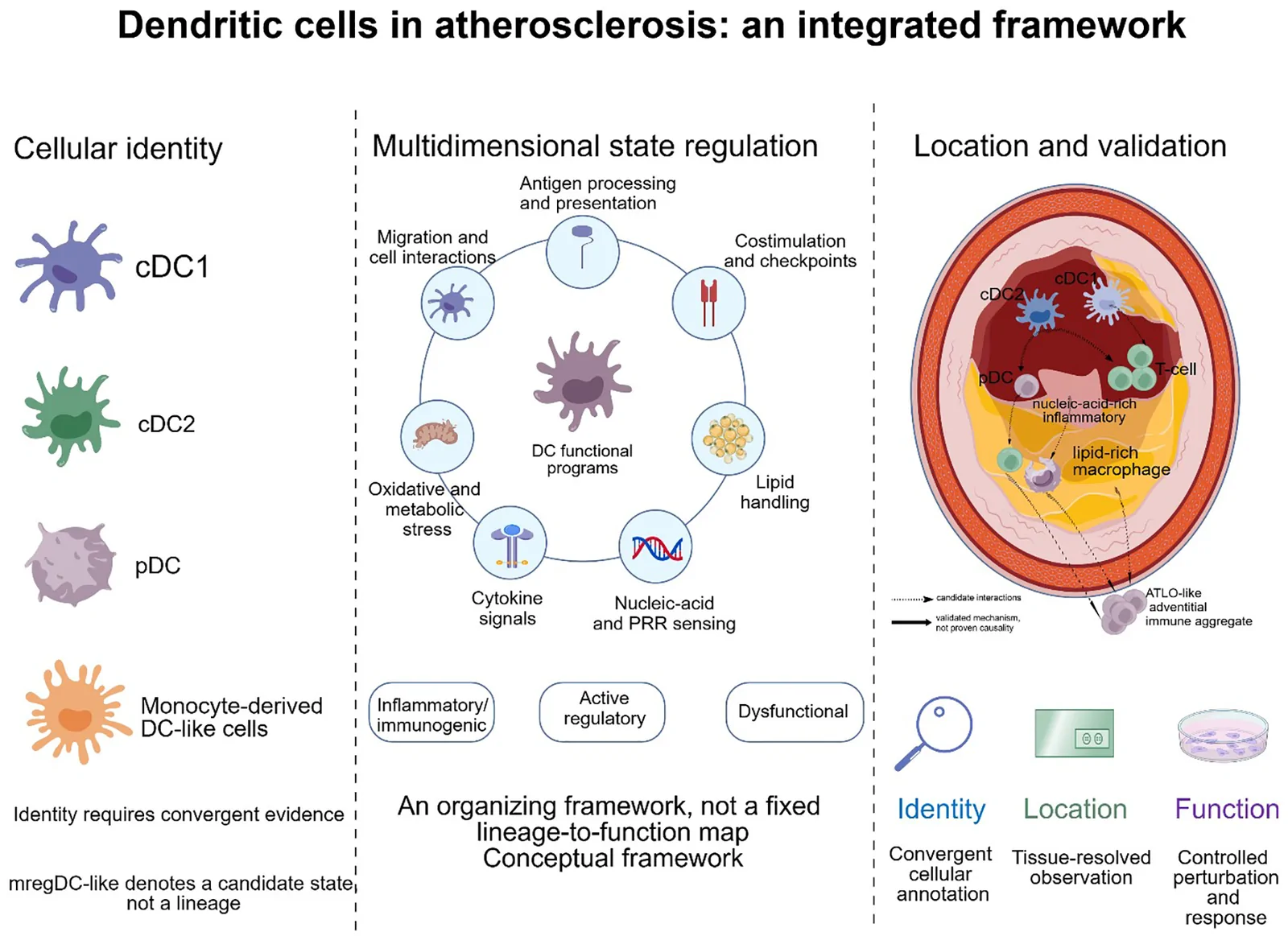
Integrated framework for interpreting dendritic-cell biology in atherosclerosis. The framework distinguishes DC identity from disease-conditioned functional state, anatomical location, and level of validation. The left panel summarizes major DC populations, including cDC1s, cDC2s, pDCs, and monocyte-derived DC-like cells, while recognizing mregDC-like cells as a candidate state rather than a distinct lineage. The central panel highlights key processes shaping DC function, including antigen presentation, costimulatory and checkpoint signaling, lipid and metabolic regulation, innate sensing, cytokine responses, migration, and cellular interactions. The right panel links candidate plaque interactions to a validation hierarchy integrating cellular annotation, tissue-resolved localization, and functional testing. Dotted arrows indicate proposed interactions, whereas solid arrows denote experimentally supported mechanisms that do not necessarily establish causality in human plaques.

Structured searches of PubMed, Web of Science Core Collection, and Scopus were conducted from database inception to July 10, 2026, using terms related to atherosclerosis, DC biology, single-cell and spatial profiling, and therapeutic modulation. Priority was given to English-language studies providing lineage-resolved, functional, spatial, cross-species, or therapeutic evidence, with direct functional evidence weighted more heavily than transcriptomic associations or mechanistic inference. Differences in species, disease stage, tissue compartment, cell-identification strategy, and experimental model were considered when reconciling conflicting findings. As the review was not conducted under a prospectively registered systematic-review protocol, it is presented as a structured narrative review.

## 2 DC-centered cellular interactions in atherosclerosis

DCs participate in atherosclerotic immunity through interactions with T cells, other antigen-presenting cells, and vascular cells. Human plaque observations and experimental studies support roles for DC-associated antigen presentation, costimulation, and chemotactic signaling in local T-cell responses (37–39). **Figure 2** summarizes the antigen-presentation, costimulatory, checkpoint, and cytokine pathways through which DCs can shape T-cell responses and vascular outcomes.

**Figure 2:**
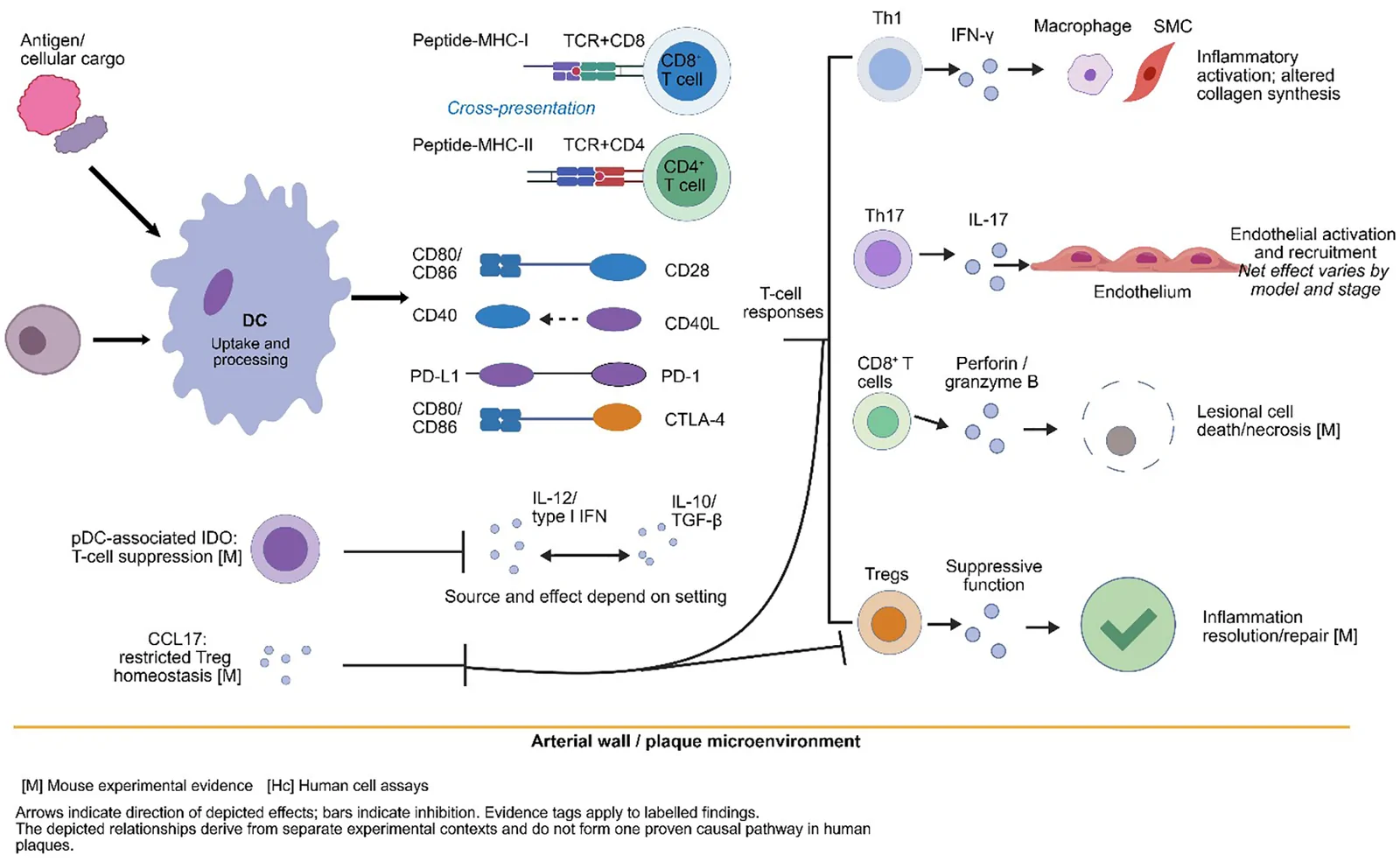
Dendritic-cell regulation of T-cell responses and candidate vascular effects. DCs present antigen through MHC-I and MHC-II and integrate costimulatory, inhibitory, checkpoint, and cytokine signals that shape T-cell responses. The schematic also shows pDC-associated IDO activity, CCL17-associated regulation of Treg homeostasis, and downstream effects on vascular inflammation, injury, and repair. Arrows indicate direction of effect and bars inhibition; [M] and [H] denote mouse experimental evidence and human cell assays. Relationships from separate studies do not constitute a single established causal pathway in human plaques.

### 2.1 Antigen presentation and DC–T-cell instruction

DCs regulate T-cell responses by integrating antigen presentation with costimulatory, inhibitory, and cytokine signals (19–21). MHC class I-mediated cross-presentation supports CD8^+^ T-cell responses, whereas MHC class II-mediated presentation contributes to CD4^+^ T-cell activation and differentiation. These responses are further shaped by antigen availability, costimulatory strength, DC functional and metabolic state, and regulatory signals (27, 40).

Atherosclerotic plaques contain T-cell subsets with distinct and sometimes opposing functions (41, 42). Th1 cells are the CD4^+^ T-cell subset most consistently associated with proatherogenic activity (43, 44). Through interferon gamma (IFN- γ) production, they activate macrophages, amplify local inflammation, and repress collagen synthesis in vascular smooth muscle cells (SMCs), a process relevant to plaque stability (45). Th17/interleukin-17 (IL-17) responses can promote endothelial activation and leukocyte recruitment, although their net effects remain context dependent and vary across experimental systems (46, 47). By contrast, regulatory T cells (Tregs) generally suppress effector T-cell responses, limit plaque inflammation, and support lesion stability through interleukin-10 (IL-10), transforming growth factor beta (TGF- β), and contact-dependent mechanisms (48–52). CD8^+^ T cells also contribute to vascular immune responses and may participate in tissue injury (11–14). These T-cell outcomes should be distinguished from direct evidence that a particular DC population causes plaque progression or stabilization.

DC–T-cell communication is reciprocal (53). DC-derived IL-12 favors Th1 differentiation, whereas IL-10 and TGF- β-related signaling can support regulatory responses (54). Activated T cells, in turn, can modify DC maturation and costimulation through cytokine and cell-contact signals (55, 56). In human unstable plaques, activated DCs have been observed near activated T cells, together with CCL19, CCL21, and CD86 expression (37, 57). These findings are consistent with local reciprocal signaling but do not establish antigen-specific interaction. Reduced T-cell stimulation likewise cannot distinguish active tolerance from impaired DC function without additional functional evidence.

### 2.2 DC interactions with other immune and vascular cells

DCs function within an antigen-presenting network that includes monocytes, macrophages, and B cells (58, 59). In human plaques, lipid-laden macrophages can present CD1-restricted lipid antigens to T cells (60), underscoring that antigen presentation is not unique to DCs. Overlapping phenotypic markers further complicate attribution of local immune effects to specific myeloid populations. B cells link antigen presentation to humoral immunity and contribute to organized lymphoid niches in atherosclerosis. Their coexistence with DCs in these sites may facilitate local immune interactions but does not by itself demonstrate a direct functional relationship.

Vascular cells influence both DC recruitment and the consequences of local immune activation. Endothelial adhesion and migration pathways contribute to DC trafficking (61, 62). Effects on SMCs may also be indirect; for example, Th1-associated IFN- γ suppresses SMC collagen synthesis (45), while candidate DC–CD8^+^ T-cell interactions may contribute to vascular injury. Direct DC effects, lymphocyte-mediated effects, and spatial associations should therefore be interpreted separately.

### 2.3 Microenvironmental regulation of DC communication

Modified lipoproteins, inflammatory cytokines, oxidative stress, and cell-death products can alter DC antigen handling and communication with neighboring cells (22, 23, 63, 64). These effects vary with the experimental conditions and DC population examined. Lipid accumulation, for example, has been associated with altered maturation or reduced T-cell stimulation in some studies, whereas others report preserved antigen processing and priming under hypercholesterolemic conditions (65, 66). Hypoxia and necrotic material may provide additional local stresses, although findings from other plaque myeloid cells cannot be directly extrapolated to lineage-defined DCs.

## 3 DC lineages and disease-conditioned functional states

### 3.1 Developmental identity and dimensions of functional regulation

DCs are classified primarily by developmental origin and lineage-associated transcriptional programs rather than by individual surface markers, whose expression varies with species, tissue processing, inflammation, and disease stage (67). This review considers cDC1s, cDC2s, and pDCs as developmentally defined populations, whereas monocyte-derived DC-like cells are treated separately because acquisition of DC-associated phenotypic or antigen-presenting features does not establish equivalence to a conventional DC lineage.

cDC1 development depends largely on interferon regulatory factor 8 (IRF8)- and basic leucine zipper ATF-like transcription factor 3 (BATF3)-associated transcriptional programs (68). In atherosclerosis models, IRF8-dependent DCs and BATF3-dependent antigen-presenting populations have been manipulated using distinct strategies, with differing effects on adaptive immunity and lesion outcomes (24, 26). cDC1s specialize in major histocompatibility complex (MHC) class I-mediated cross-presentation of exogenous antigens to CD8^+^ T cells, although this specialized capacity should not be taken to imply that cross-presentation alone determines their functional role in atherosclerosis.

cDC2s are more heterogeneous. Their development is associated with interferon regulatory factor 4 (IRF4)-dependent programs, although cDC2 identity cannot be defined by a single transcription factor or phenotypic marker. Through MHC class II-mediated antigen presentation, cDC2s regulate CD4^+^ T-cell activation and polarization (69). Their cytokine production, costimulatory activity, metabolic state, and migratory behavior are sensitive to local signals, enabling different functional responses across tissues and inflammatory settings. Single-cell studies further indicate that the conventional CD1c-associated/cDC2 compartment contains substantial transcriptional and developmental heterogeneity (70).

A transcription factor 4 (TCF4)-linked program supports pDC development and identity. pDCs specialize in sensing pathogen- and self-derived nucleic acids and can rapidly produce type I interferons after activation, although inflammation may alter their phenotype and complicate their identification. Their functional output should therefore be distinguished from developmental identity and evaluated according to the stimulus, anatomical compartment, and measured immune or vascular response.

In contrast, monocyte-derived DC-like cells do not constitute a developmentally stable lineage equivalent to conventional DCs. Recruited monocytes can acquire DC-associated phenotypic and antigen-presenting features in response to inflammatory differentiation signals, including granulocyte-macrophage colony-stimulating factor (GM-CSF). Depending on the local cytokine and tissue environment, these cells may acquire antigen-presenting capacity, produce inflammatory mediators, or participate in immune regulation. Because their transcriptional and phenotypic profiles overlap with those of macrophages and conventional DCs, surface phenotype alone is insufficient for identification. Developmental origin and acquired DC-like phenotype should therefore be considered separately, with classification integrating developmental evidence, transcriptomic identity, and protein-level measurements whenever possible (20, 71, 72).

Cross-species comparisons should integrate developmental origin, transcriptional identity, phenotype, and function. Although cDC1 development and cross-presentation are relatively conserved, cDC2- and pDC-associated populations show greater heterogeneity between mice and humans. Modified lipoproteins can alter DC maturation and immune function, whereas plaque hypoxia alters lipid metabolism and inflammatory programs in other lesional myeloid cells (63, 64, 73). Whether these pressures generate equivalent lineage-resolved DC states in mouse and human plaques remains to be established.

Accordingly, the functional comparisons below consider antigen handling, costimulatory and checkpoint signals, chemokine and cytokine output, lipid and redox metabolism, migration, and interactions with neighboring cells. These dimensions are evaluated as complementary determinants of DC state rather than as lineage-defining characteristics, and evidence derived from one dimension should not be assumed to establish the others. Representative developmental evidence and identification features of conventional DCs are summarized in **Table 1**.

**Table 1:** Developmental evidence and representative identification features of conventional dendritic cells.

Population	Developmental evidence	Representative identification features	Functional emphasis
Mouse cDC1	IRF8–BATF3-associated developmental program; perturbation specificity depends on the model	XCR1; CD8 α in lymphoid populations or CD103 in appropriate tissue populations, interpreted with a broader DC phenotype	Specialized MHC-I cross-presentation; also capable of CD4^+^ T-cell instruction (25, 26, 68, 71, 74)
Human cDC1	Human developmental and IRF8-deficiency studies support an IRF8-sensitive developmental trajectory	CD141 together with CLEC9A; IRF8-associated transcriptional identity	Cross-presentation-associated identity; plaque function requires separate testing (29, 75)
Mouse cDC2	IRF4 supports development or maintenance of defined tissue CD11b^+^ DC subsets; dependence is not a universal single-factor definition	CD11b/SIRP α within a DC identification panel; overlapping macrophage populations should be excluded	MHC-II-mediated CD4^+^ T-cell instruction; responses vary with tissue and stimulus (71, 76)
Human CD1c-associated DC compartment	Developmental studies distinguish heterogeneous trajectories within the CD1c^+^ compartment, including populations with different IRF8 requirements	CD1c with CLEC10A/FCER1A-associated identity; additional markers can help resolve heterogeneity in validated panels	CD4^+^ T-cell instruction; a broad CD1c-associated plaque cluster does not resolve every developmental population (29, 75–77)

### 3.2 cDC1s: antigen presentation and regulation of T-cell responses

cDC1s specialize in MHC class I-mediated cross-presentation, but their contribution to atherosclerosis is not explained by cross-presentation alone. IRF8- and BATF3-dependent perturbation studies have produced different lesion outcomes: IRF8-dependent DC depletion reduced proatherogenic adaptive immunity, whereas BATF3-deficient models showed either reduced or unchanged atherosclerosis (24–26, 78). These findings distinguish cDC1 lineage or cross-presentation capacity from the requirement for a particular pathway in a given disease model.

cDC1 activity also involves functional components beyond cross-presentation. In peptide-driven cocultures, BATF3-dependent DCs promoted Th1 differentiation through IL-12, linking cDC1-associated activity to helper-T-cell instruction (25). More recent studies identified STING-dependent cDC1 signaling that affected lesion development and Th1/CD8^+^ effector responses without altering cDC1 abundance, and implicated XCL1–XCR1 communication in cDC1–lymphocyte interactions (74, 79). STING-associated sensing, XCL1–XCR1-mediated communication, and IL-12-mediated T-cell instruction therefore represent distinct components of cDC1 function rather than one established causal pathway.

Regulatory observations provide a counterpoint to these inflammatory mechanisms. Arterial DCs show relatively high PD-L1 expression under baseline conditions (80), while transfer of oxLDL-induced apoptotic DCs increased CD103^+^ DCs and Tregs and reduced atherosclerosis (81). These findings support inhibitory or treatment-associated regulatory responses but do not establish an endogenous tolerogenic plaque cDC1 population.

Interpretation remains sensitive to the genetic strategy, disease model, and measured outcome because IRF8-, BATF3-, XCR1-, CD103-, FLT3-, and CD11c-based approaches do not manipulate identical populations (24, 26, 79). Lesion burden and immune activation should therefore be treated as related but distinct outcomes.

Human evidence remains limited. Single-cell studies identify cDC1-like populations in human atherosclerotic plaques, but do not establish antigen-specific cross-presentation, STING- or XCR1-dependent function, or causal tissue injury (82). Shared markers and sample-processing effects further complicate identification (71, 83, 84). Thus, experimental studies support several separable cDC1-associated mechanisms—antigen presentation, IL-12-mediated T-cell instruction, STING-associated sensing, XCL1–XCR1 communication, and inhibitory checkpoint activity—but their relative importance and integration in human plaques require direct functional validation.

### 3.3 cDC2s: chemokine regulation, lipid handling, and T-cell instruction

cDC2-mediated CD4^+^ T-cell instruction depends on MHC class II antigen presentation together with chemokine output, costimulatory and checkpoint signals, lipid handling, and migratory capacity. Because several relevant studies examine broader conventional or myeloid DC populations rather than lineage-resolved plaque cDC2s, these mechanisms should be interpreted as complementary dimensions of cDC2-associated function rather than as properties established for every cDC2 population.

CCL17-expressing DCs provide one experimentally supported chemokine mechanism. CCL17-associated signaling restricts Treg homeostasis and promotes experimental atherosclerosis (85, 86). However, CCL17 expression does not independently establish cDC2 lineage, and the experimentally supported CCL17–Treg circuit should not be interpreted as a lineage-exclusive human plaque cDC2 program.

Lipid handling provides a distinct functional dimension. Under hypercholesterolemic conditions, identified peripheral cDC2 subsets accumulated lipids and reactive oxygen species (ROS) and displayed activation-associated features while showing reduced naïve CD4^+^ T-cell priming (65). By contrast, broader CD11c^+^ DC populations have retained antigen processing and T-cell priming under hypercholesterolemic conditions (66). Thus, lipid accumulation, surface maturation, and antigen-presenting competence are separable outcomes rather than successive markers of one functional state. Consistent with this distinction, experimental enhancement of cholesterol efflux reduced MHC class II and CD80/CD86 expression together with CD4^+^ T-cell activation (87).

Checkpoint availability and migration provide additional, non-equivalent dimensions of DC function. In coronary artery disease, reduced PD-L1 expression on circulating myeloid DCs was associated with greater T-cell activation, while experimental enhancement of PD-L1 restrained T-cell proliferation and inflammatory cytokine production (88). Lipid exposure can also alter migratory machinery; oxLDL has been reported to reduce CCR7 expression and CCL21-dependent migration in experimental DC systems (89). These findings show that checkpoint regulation and migratory competence can change independently of antigen-presentation phenotype, but neither establishes a plaque-specific protective cDC2 state.

These mechanisms are supported at different levels of lineage resolution and should not be combined into a single cDC2 pathway. Lipid-associated impairment of T-cell priming has been measured in identified cDC2 subsets, whereas CCL17, PD-L1, cholesterol-efflux, and migration studies do not all resolve the same cDC2 population or anatomical compartment. Human evidence remains particularly limited: CCL17 associations and circulating-DC checkpoint findings do not establish that a lineage-resolved plaque cDC2 population simultaneously expresses these programs (88, 90). Thus, chemokine signaling, lipid/redox state, antigen-presentation competence, checkpoint availability, and migration represent complementary mechanisms, but a complete human plaque cDC2 functional network has not been demonstrated.

### 3.4 pDCs: nucleic-acid sensing, immune regulation, and vascular dysfunction

pDCs connect nucleic-acid sensing with type I interferon production and immune regulation (91). In atherosclerosis, protein–DNA complexes and nucleic acids released from injured tissue provide relevant activating stimuli (92). In ApoE-deficient mice, pDC depletion reduced lesion development and T-cell activation, while human plaque studies linked pDC-associated IFN- α activity to enhanced cytotoxic T-cell function (28, 93). These findings support an inflammatory pDC program in defined settings, but depletion does not identify type I interferon as the sole mediator and the human observations do not establish a universal IFN-high plaque-pDC state.

Protective mechanisms have also been demonstrated experimentally. In Ldlr^-/-^ mice, pDC depletion increased plaque T-cell accumulation and lesion development, while inhibition of indoleamine 2,3-dioxygenase (IDO) abolished the suppressive effect of lymphoid-tissue pDCs on T-cell proliferation (94). A separate study linked IDO-expressing aortic pDCs to Treg induction and atheroprotection (27). These findings support IDO-associated immune regulation, but IDO expression alone should not be taken as evidence of a tolerogenic phenotype without a corresponding regulatory functional outcome.

The vascular consequences of pDC activation extend beyond immune-cell regulation. In db/db mice, isolated pDCs impaired ATP-stimulated endothelial nitric oxide synthase phosphorylation in cultured endothelial cells and reduced endothelium-dependent arterial relaxation. Antibody-mediated pDC depletion improved endothelial function, while diabetic pDCs showed increased inflammatory and endoplasmic-reticulum (ER)-stress-associated markers (95). These findings provide experimental evidence that pDCs can contribute to endothelial dysfunction in a cardiometabolic setting, although they do not establish an equivalent mechanism in human atherosclerotic plaques.

A complementary vascular mechanism has been reported in renovascular hypertension. In mice with two-kidney, one-clip hypertension, pDC depletion improved microvascular function and reduced blood pressure, inflammation, and oxidative stress, together with changes in ER-stress-, autophagy-, and mTOR-related measures (96). This evidence extends the potential vascular effects of pDCs to a hypertensive setting and links them to inflammatory, oxidative, and cellular-stress pathways.

These vascular studies broaden the disease contexts in which pDC activity may be relevant, but they do not demonstrate pDC-mediated plaque destabilization in humans. The diabetic experiments involved mouse pDCs and endothelial-cell systems rather than patient-derived plaque pDCs, whereas systemic immune and hemodynamic effects in the hypertension model complicate separation of direct endothelial actions from indirect vascular benefit. Endothelial dysfunction, systemic blood-pressure regulation, immune modulation, and plaque remodeling should therefore be treated as distinct outcomes.

Differences in stimulus, anatomical compartment, depletion strategy, and disease model remain important when comparing pDC studies. Antibody-mediated depletion can affect circulating, lymphoid, and vascular populations simultaneously, and plaque burden, T-cell regulation, endothelial function, and blood pressure represent distinct outcomes. Because pDCs are rare and phenotypically sensitive to inflammation, human studies additionally require careful lineage identification and tissue-resolved validation.

Human evidence remains limited. Studies in coronary artery disease have reported altered circulating pDC abundance or function (97–99), but circulating measurements do not establish intraplaque localization or mechanism. Overall, current evidence supports several separable pDC-associated functions—type I interferon-linked inflammation, IDO-associated immune regulation, and vascular dysfunction in cardiometabolic and hypertensive models—whose relative importance depends on stimulus, compartment, and disease setting. Their contribution to human plaque progression or destabilization requires direct tissue-resolved and functional validation.

### 3.5 Monocyte-derived DC-like cells: inflammation-induced state plasticity rather than a fixed lineage

Monocyte-derived DC-like cells arise when recruited monocytes acquire DC-associated properties under inflammatory conditions. Unlike cDC1s and cDC2s, they should not be defined as a stable lineage solely by CD11c and MHC class II expression. In atherosclerotic aortas, CD11b^+^ DC-like cells may express F4/80 or acquire CD64, indicating substantial overlap with monocyte–macrophage populations (100). Their identification therefore requires evidence of developmental origin in addition to phenotypic and antigen-presenting features.

Experimental studies support recruitment of inflammatory monocytes and acquisition of DC-like antigen-presenting features in atherosclerosis (101, 102). DC migration is regulated by endothelial interactions and chemokine-receptor programs (61, 62), while local APC–T-cell interactions can activate adaptive immunity within the arterial wall (39, 102). These observations support related processes but do not establish a continuous monocyte-to-DC differentiation and migration pathway within plaques.

Human blood monocyte-derived DC experiments provide complementary evidence for lipid-responsive functional states. OxLDL increased maturation-associated molecules and promoted IFN- γ- and IL-17-associated T-cell responses, whereas statin treatment reduced CD80/CD83/CD86 and inflammatory cytokine production and increased IL-10, TGF- β, and FoxP3-associated regulatory responses (103, 104). Importantly, T cells generated with atorvastatin-treated DCs suppressed autologous responder T-cell proliferation in a secondary coculture assay (103). This functional suppression provides stronger evidence of an induced regulatory response than reduced maturation or T-cell stimulation alone.

The functional effect of lipid exposure is not uniform: oxLDL can promote maturation in human monocyte-derived DCs, whereas broader DC populations may retain antigen processing and CD4^+^ T-cell priming under hypercholesterolemic conditions (64, 66). Reduced stimulatory capacity should therefore not be equated with active tolerance unless accompanied by functional evidence such as regulatory T-cell activity or suppression of responder-cell proliferation.

Terminological and methodological differences remain important. DCs generated from peripheral-blood monocytes *in vitro* are not equivalent to DC-like populations identified within arterial tissue, even when plaque-derived T cells are used in coculture experiments (103, 104). Phenotypic overlap among conventional DCs, monocyte-derived cells, and macrophages further complicates tissue annotation (20, 71, 72). Human single-cell and multimodal studies likewise reveal monocyte–macrophage continua rather than establishing a discrete endogenous plaque moDC lineage (105, 106). Thus, culture experiments demonstrate modifiable human DC–T-cell functions, but they do not establish the developmental origin or in-plaque behavior of an equivalent human moDC-like population. **Table 2** separates phenotypic observations from directly assessed functional outcomes and their evidence limits.

**Table 2:** Phenotypic observations, functional evidence, and interpretation of DC-associated responses in atherosclerosis and related vascular models.

Cell population and experimental setting	Perturbation or exposure	Phenotypic or molecular observations	Directly assessed functional or disease outcome	Supported interpretation and limit
cDC1s in murine atherosclerosis	Lineage-directed depletion; STING-related and XCL1–XCR1 interventions in separate studies	Altered cDC1-associated immune signaling	Changes in T-cell responses and lesion development; earlier BATF3-based studies yielded differing lesion outcomes (24–26, 74, 78)	Supports disease-promoting cDC1 activity in specified models, but not a universal phenotype or a single experimentally established STING–XCR1 pathway (79).
	DC-restricted or broader DNGR-1 deletion under moderate hypercholesterolemia; separate testing of bone-marrow-derived IL-10 dependence	Increased IL-10 expression	Reduced lesional inflammation and atherosclerosis; protection was abolished without bone-marrow-derived IL-10 (107)	Supports IL-10-dependent atheroprotection after DNGR-1 deletion. It does not establish that all protective IL-10 originates from DCs or define an endogenous tolerogenic cDC1 subset.
Splenic $\text{CD}103^{+}$ DC-associated response in hyperlipidemic mice	Transfer of oxLDL-induced apoptotic DCs	Increased CD103 + splenic DCs and Tregs	Reduced atherosclerosis after the treatment intervention (81)	Supports a treatment-associated protective response; these abundance changes alone do not establish the suppressive function or necessity of the expanded DC population.
CCL17-expressing DC-associated circuit in experimental atherosclerosis	CCL17 deficiency/blockade and investigation of the CCR8–CCL3–CCR1 circuit	Altered chemokine signaling and Treg homeostasis	Changes in Treg regulation and lesion development (85, 86)	Supports a disease-promoting chemokine circuit. CCL17 expression is not sufficient to assign cDC2 lineage, and pathway inhibition does not itself establish DC-intrinsic tolerance.
Peripheral cDC2 subsets in hypercholesterolemic mice	*In situ* lipid loading	Lipid and ROS accumulation; increased activation-associated features	Reduced naïve T-cell priming (65)	Demonstrates impaired stimulatory function under the tested conditions; it does not demonstrate active suppression or tolerogenic conversion.
pDCs in inflammatory atherosclerosis studies	Depletion or nucleic-acid-associated stimulation in mice; IFN- α-related investigation in human plaque systems	Type I IFN-associated activity	Altered murine lesions and T-cell activation (28, 92); enhanced cytotoxic T-cell function in the human plaque study (93)	Supports inflammatory mechanisms in the respective systems; depletion alone does not isolate an IFN-specific mechanism, and human functional observations do not establish clinical causality.
Lymphoid and aortic pDCs in regulatory atherosclerosis studies	pDC depletion, IDO inhibition, and antigen-presentation experiments in distinct models	IDO expression; altered aortic Treg abundance and IL-10	IDO blockade reversed lymphoid-pDC suppression of T-cell proliferation; aortic pDCs expanded antigen-specific Tregs; depletion aggravated murine disease (27, 94)	Supports experimentally tested regulatory functions. Lymphoid suppression and aortic Treg responses represent distinct evidence chains; human pDC–Treg colocalization is not equivalent to the murine functional experiments.
pDCs in diabetic and renovascular-hypertensive mice	pDC exposure to endothelial/arterial preparations; systemic antibody-mediated depletion	Altered endothelial eNOS phosphorylation and stress-associated measures	Changes in endothelial relaxation; depletion improved vascular function, with blood-pressure reduction in hypertension (95, 96)	Supports vascular involvement outside classical atherosclerosis models. Endothelial dysfunction is not evidence of impaired DC antigen presentation or plaque destabilization.
Human blood monocyte-derived DC cultures	oxLDL exposure and statin treatment	Reduced maturation markers; increased IL-10 and FoxP3-positive T cells after treatment	Treatment-conditioned T cells suppressed autologous responder T-cell proliferation in a secondary assay (103)	Demonstrates an induced functional regulatory response *in vitro*, rather than a naturally occurring suppressive plaque DC subset.
Human blood monocyte-derived DC cultures	oxLDL exposure and PCSK9 silencing	Altered HLA-DR, costimulatory molecules, scavenger receptors, and cytokines	Reduced T-cell activation and altered polarization, including experiments with plaque-derived T cells (104)	Supports modifiable DC–T-cell responses in culture; plaque T-cell origin does not establish plaque origin of the DCs.

### 3.6 Single-cell evidence for DC states: cross-study synthesis and functional interpretation

#### 3.6.1 Cross-study identification of human DC populations

Human plaque studies differ in sampling, assay design, and annotation depth. Fernandez et al. identified CD1c DC and pDC categories in carotid plaques, with antibody-informed reference annotation from paired plaque–blood CITE-seq supporting cellular identification (30). Depuydt et al. identified a CD1C/CLEC10A/FCER1A-expressing DC population in carotid plaques, complemented by chromatin-accessibility measurements (77). Bashore et al. reported CD303-associated pDC and CD141-associated conventional DC populations using CITE-seq and scRNA-seq, whereas Traeuble et al. resolved cDC1, cDC2, and pDC programs and supported atlas-level annotations using expert consensus and independent surface-protein measurements (29, 105).

Across these studies, CD1c-associated conventional DC populations and pDC annotations recur more consistently than a uniform fine-grained subtype inventory. Differences in cDC1/cDC2 resolution can reflect assay depth, reference mapping, vascular bed, cell enrichment, and rare-cell recovery rather than incompatible biology (29, 30, 77, 105). Thus, current human datasets support reproducible DC-associated identities more strongly than shared disease-specific functions. **Table 3** compares the populations identified, anatomical information available, validation approach, and main limitations of these datasets.

**Table 3:** Comparative human single-cell and spatial evidence relevant to DC identity and state.

Study/original reference	Sampling and assay scope	Reported DC populations or programs	Spatial information	Contribution to validation	Main boundary of interpretation
Fernandez et al., 2019 (30)	Carotid plaque/blood study; 46 patients across distinct cohorts. Broad immune CyTOF cohort: 15 patients; paired plaque–blood CITE-seq reference: one patient; plaque scRNA-seq: six patients	CD1c DC and pDC categories supported by antibody-derived-tag reference annotation and label transfer	Dissociated assays do not retain native DC position	Antibody-informed identification; complementary cytometric profiling	Assays cover different subsets of patients. A single CITE-seq reference is not independent protein validation of each transcriptomic sample; main analyses emphasize T cells/macrophages
Depuydt et al., 2020 (77)	Carotid endarterectomy plaques from 18 patients; all-viable-cell scRNA-seq, with complementary scATAC-seq	My.3 DC population: CD1C, CLEC10A, FCER1A and prominent class-II HLA expression	Dissociated assays do not provide a DC-specific tissue map	Chromatin accessibility complements RNA-based identity	Chromatin activity is not measured antigen presentation or cytokine secretion; broad tissue histology does not independently validate DC subtype function
Zernecke et al., 2023 (71)	Integration of 12 mouse datasets and two human studies comprising 11 patients: four coronary and seven carotid samples	Mouse Xcr1-associated cDC1, Cd209a-associated cDC2, and Ccr7/Fscn1 mature/mreg-DC programs; related human transcriptomic populations	Integration does not restore native spatial coordinates	Cross-dataset and cross-species transcriptomic correspondence	Reused cohorts do not add independent subjects; mouse markers and regulatory labels cannot automatically be transferred as human lineage or functional proof
Bashore et al., 2024 (105)	21 carotid patients: six CITE-seq and 15 scRNA-seq; integrated analysis with protein prediction	CD303-associated pDC and CD141-associated conventional DC populations	No native DC localization from dissociated profiling	Measured RNA/protein information in CITE-seq samples supports annotation; sciPENN predicts protein information for scRNA-seq samples	Measured and predicted proteins are different evidence; broad conventional DC annotation does not resolve all cDC subtypes
Lai et al., 2025 (31)	Human plaque scRNA-seq and Stereo-seq with histological characterization; analysis focused on plaque TLO-like regions	LAMP3-associated mature DC signatures	Signatures mapped to plaque TLO-like regions	Tissue localization and neighborhood context	Spatial signatures do not determine developmental origin, cognate antigen presentation, or immune suppression; results do not establish a universal plaque distribution
Traeuble et al., 2025 (29)	Integrated carotid, coronary and femoral plaque atlas; reference mapping tested on the Bashore dataset	cDC1: CLEC9A/IRF8; cDC2: CD1C/CLEC10A/FCER1A; pDC: TCF4/CLEC4C/TLR7/TLR9	Dissociated atlas does not establish native niches	Expert-label agreement and CITE-seq surface-protein validation of atlas-level annotations	Atlas-level validation is not independent functional validation of every DC subtype; the mapped Bashore cohort was subsequently incorporated, so its cells must not be double-counted

#### 3.6.2 Maturation programs and anatomical context

Cross-dataset integration can establish recurrence of molecular programs across samples but does not create independent biological subjects. Integrated analyses have identified mature CCR7/FSCN1-associated DC programs, including mregDC-like signatures, together with transcriptionally related human populations (71). These observations support a recurring maturation-associated program but do not establish that the corresponding populations share a developmental origin or suppressive function.

Spatial data provide a complementary layer of evidence. Lai et al. combined single-cell profiling and Stereo-seq to map LAMP3-associated mature DC signatures to plaque tertiary lymphoid organ-like regions (31). This provides anatomical context that dissociated-cell datasets cannot retain, but spatial localization alone does not identify the cognate antigen, responding T-cell clone, or direction of the immune effect.

Lineage-associated genes and maturation-associated programs should therefore be interpreted on separate axes. CCR7-, FSCN1-, or LAMP3-associated mature/mregDC-like programs represent candidate maturation states rather than additional developmental lineages, and their detection does not establish fixed plaque localization or regulatory function.

#### 3.6.3 Functional interpretation of candidate states

Coexpression of activation and inhibitory molecules is insufficient to establish tolerance. Reduced T-cell stimulation may result from defective antigen processing, reduced viability, or metabolic constraints rather than active regulation. A tolerogenic interpretation therefore requires functional evidence such as suppression of effector responses, induction of functional Tregs, regulatory cytokine activity, or mechanism-directed checkpoint dependence.

The lineage–state–location–validation framework asks four complementary questions: which cell is identified, which program is measured, where it occurs, and which function has been tested. Validation is claim-specific: protein measurements strengthen cellular identification, spatial assays establish anatomical relationships, and perturbations test functional dependence. More molecular modalities do not by themselves establish causality. The priority is therefore to connect reproducible human DC programs to measured immune or vascular effects rather than assign function from transcriptomic annotation or spatial proximity alone.

## 4 Dendritic cell states and locations in atherosclerosis

### 4.1 Atherosclerotic plaques as topological immune ecosystems

Single-cell profiling has revealed substantial immune-cell heterogeneity in atherosclerotic tissue, including rare DC populations (71, 108). Spatial and multimodal studies further show that plaque cell states are organized within distinct tissue environments (30, 31). However, these findings do not establish a fixed correspondence between individual DC lineages and anatomical compartments, nor do they demonstrate that local niches generate specific DC states. The spatial organization discussed below therefore distinguishes observations in human tissue from experimentally supported mechanisms and candidate niche-dependent functions.

The fibrous cap consists primarily of smooth muscle cells (SMCs) and collagen-rich extracellular matrix, whereas adjacent shoulder regions experience greater mechanical stress and inflammatory exposure (109). Oxygen availability decreases in deeper regions of advanced plaques, where hypoxic zones have been demonstrated experimentally (73, 110). These regions can also contain cholesterol crystals, oxidized lipids, cellular debris, extracellular nucleic acids, and other damage-associated signals. The adventitia forms a distinct immune compartment in which persistent inflammation may promote artery tertiary lymphoid organs (ATLOs) containing B-cell follicles, T-cell zones, high endothelial venules, and antigen-presenting cells (32, 33, 111).

DCs in different plaque compartments encounter distinct combinations of metabolic stress, antigens, cytokines, mechanical forces, and neighboring cells. These conditions may promote divergent states within a lineage or shared programs across lineages but do not define compartment-specific DC identities. Spatial transcriptomics can link transcriptional programs to plaque anatomy, while multiplexed imaging can refine cellular identification and localize candidate interactions (31). **Table 4** summarizes the available spatial evidence and validation gaps, and **Figure 3** distinguishes observed relationships from proposed mechanisms.

**Table 4:** Spatial evidence, candidate functions, and validation gaps for dendritic cells in atherosclerotic tissue.

Anatomical compartment or spatial question	Available observations and evidence source	Functional interpretation	Principal validation gap
Fibrous cap and shoulder: activated DC–T-cell associations	Human histology identifies CD83-positive DCs in unstable plaque shoulders, with CCL19/CCL21 expression and close contact with activated T cells (37).	Supports an activated DC-containing cellular neighborhood. A local ${cDC1–CD8}^{+}$ T-cell pathway remains a candidate mechanism rather than an established explanation for these observations.	Modern lineage assignment, antigen-specific cross-presentation, and a causal link to SMC injury or cap failure.
Plaque shoulder: pDC-associated IFN activity	A human carotid study identified IFN- α-producing pDCs in shoulder regions. Functional experiments linked IFN- α to TRAIL induction on ${CD4}^{+}$ T cells and vascular SMC killing (93).	Supports a pDC-associated inflammatory mechanism without establishing a universal pDC niche or an *in vivo* causal sequence leading to plaque rupture.	Reproducibility across patients and vascular beds; spatially resolved causal validation in intact lesions.
Lipid-rich and deep plaque regions	Experimental plaque studies demonstrate hypoxia (73). Separate human *in vitro* and murine studies show that lipid exposure can promote DC maturation or preserve antigen presentation and $\text{CD}4^{+}$ T-cell priming (64, 66).	Lipid-related responses are heterogeneous; hypoxia or lipid accumulation alone does not establish DC dysfunction.	Direct linkage of local oxygen and lipid exposure to lineage-defined human DCs and separately measured antigen processing, migration, and T-cell stimulation.
Necrotic-core boundary: proposed pDC localization	pDC nucleic-acid sensing and IFN-associated effects provide mechanistic context (92, 93), but the cited evidence does not establish preferential pDC localization at the human core–cap interface.	Local nucleic-acid sensing is a candidate mechanism; an IFN-high pDC niche at this boundary remains a spatial hypothesis.	Demonstration of pDC identity, local stimulus exposure, IFN production, and functional consequences at the proposed interface.
Lipid-rich or necrotic regions: proposed maturation-associated and immunoregulatory DC programs	Available evidence does not directly validate an mregDC-like population enriched at a hypoxic–necrotic interface; no direct localization reference has yet established this compartment-specific population.	This remains a question for spatial mapping rather than an established compartment-specific identity or evidence of tolerogenic function.	Lineage-resolved mapping across lesions and functional distinction between active immune regulation and impaired T-cell stimulation.
Adventitial ATLOs	Human coronary histology identifies adventitial tertiary lymphoid structures (122). In aged Apoe^-/-^ mice, ATLOs support antigen presentation and local T-cell responses, including regulatory responses (117).	Supports organized adventitial immunity, but does not establish preferential cDC2 enrichment or a cDC2-dominated priming–effector circuit.	Human DC-subset attribution, local functional contribution, and the direction and timing of connections with intimal plaques and draining lymphoid tissues.
Intraplaque TLO-like structures	Human spatial single-cell analysis identifies plaque TLO-like structures with prominent B-cell organization (31).	Establishes a plaque-associated lymphoid context that should be distinguished anatomically from adventitial ATLOs; it does not by itself establish a DC-subset-specific function.	Direct DC identification and functional assessment within these structures, including comparison across lesions and vascular beds.

**Figure 3:**
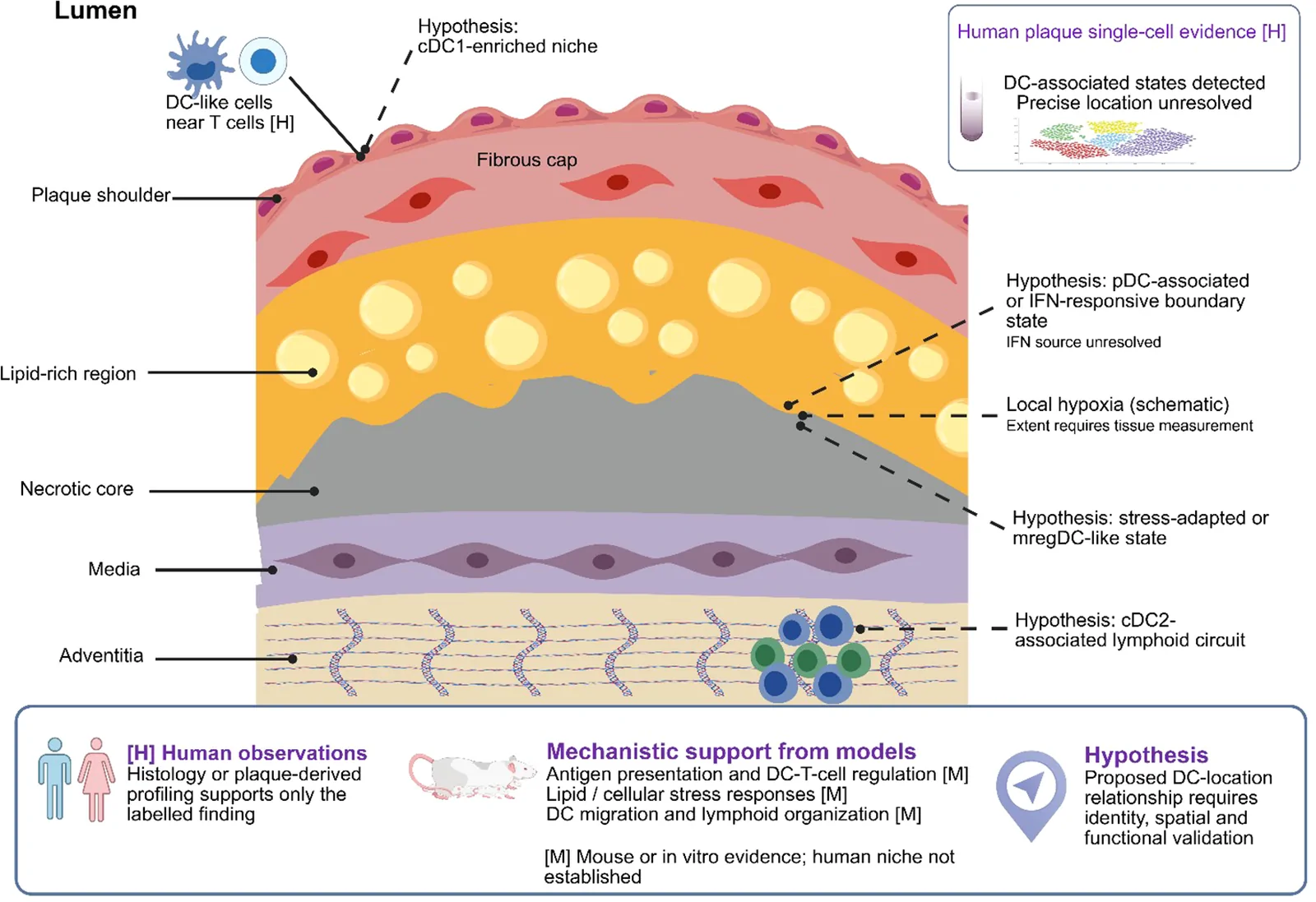
Spatial organization of dendritic-cell observations and proposed niches in atherosclerotic plaques. Human histology and plaque profiling identify DC-like cells and DC-associated states but often do not resolve their precise location or function. Dashed lines indicate proposed spatial associations, including cDC1-enriched, pDC/interferon-responsive, stress-adapted or mregDC-like, and cDC2-associated adventitial niches. Model-based evidence supports selected mechanisms but does not establish the corresponding human niches, which require validation of cellular identity, localization, and function.

### 4.2 Fibrous cap and plaque shoulder: DC–T-cell associations and a candidate cDC1–CD8^+^ T-cell axis

The fibrous cap consists mainly of SMCs embedded in collagen-rich extracellular matrix and separates the thrombogenic necrotic core from circulating blood. The adjacent shoulder regions experience substantial mechanical stress and inflammatory activity and are therefore particularly vulnerable to rupture. DCs in superficial plaque regions may encounter oxLDL and plaque-associated antigens, but the precise exposure, lineage identity, and antigen composition of individual DCs have not been resolved spatially (64, 103, 104).

Human plaque studies identify vascular DCs and activated DC–T-cell aggregates in unstable shoulder regions (37, 112). These observations support a local DC–T-cell association, but do not establish cDC1 lineage identity, antigen-specific cross-presentation, or a causal contribution to vascular injury. A candidate mechanism is that local cDC1-mediated cross-presentation activates or restimulates cytotoxic CD8+ T cells, whose perforin, granzymes, IFN- γ, and tumor necrosis factor may injure endothelial cells and SMCs.

Because SMCs maintain fibrous-cap collagen, such a pathway could plausibly link T-cell activation to SMC injury and cap thinning. Demonstrating this mechanism in human plaques would require evidence connecting lineage-defined cDC1s, antigen-specific T-cell responses, and local SMC injury or cap failure. The proposed cDC1–CD8^+^ T-cell axis should therefore be considered a mechanistically supported hypothesis rather than an established cause of human plaque instability.

### 4.3 Lipid-rich and necrotic regions: microenvironmental stress and candidate DC states

As plaques progress, lipid-rich regions merge with necrotic areas in which oxidized lipids, cholesterol crystals, dead-cell remnants, extracellular nucleic acids, and other damage-associated signals accumulate (113). Direct studies demonstrate hypoxic zones in deep regions of experimental plaques and relate them to diffusion distance and cellular oxygen consumption (73, 110). These observations characterize the local microenvironment but do not establish which lineage-defined DC populations occupy these regions or how they respond *in situ*. In human plaques, the spatial relationships among DC identity, oxygen exposure, lipid accumulation, and functional state remain insufficiently resolved.

Hypoxia-associated signaling and altered lipid handling provide plausible mechanisms through which these microenvironments may influence DC function. Lipid uptake, cholesterol esterification and efflux, endoplasmic-reticulum stress, and autophagic or lysosomal activity can affect antigen-presenting cells, while hypoxia may alter metabolic and inflammatory programs. However, these processes should not be assembled into a single established pathway from local hypoxia and lipid accumulation to DC dysfunction in human plaques. Antigen processing, migration, cytokine production, and T-cell priming should instead be assessed as distinct functional outcomes in lineage-defined cells.

Foam cell-like DCs have been identified histologically in atherosclerotic plaques, but morphology alone cannot reliably distinguish lipid-laden DCs from macrophages or establish a causal pathway from cholesterol accumulation to DC dysfunction. Experimental studies instead show heterogeneous responses: modified lipoproteins can promote maturation, while cholesterol-loaded DCs may retain antigen processing and CD4^+^ T-cell priming (64, 66). Mechanistic evidence remains derived largely from murine and *in vitro* systems, and its relevance to lineage-defined human plaque DCs is not yet established.

Nucleic-acid sensing by pDCs provides a candidate mechanism linking damage-associated signals to local inflammatory activity. Extracellular nucleic acids and nucleic acid-containing immune complexes can activate pDCs through Toll-like receptors 7 and 9 and promote interferon- α-associated responses (92, 114). Such signaling may amplify inflammation and cytotoxic activity (28, 93). However, available evidence does not establish preferential localization of IFN-producing pDCs at the human necrotic-core boundary or a causal contribution to injury at the core–cap interface. Indeed, IFN- α-producing pDCs have also been identified in human plaque shoulder regions (93).

Maturation-associated or mregDC-like programs may also be relevant to lipid-rich and necrotic regions, but their regional enrichment remains a spatial hypothesis. Demonstrating such an association requires lineage-resolved mapping across lesions, followed by functional testing to distinguish active immune regulation from impaired T-cell stimulation (115).

Taken together, lipid-rich and necrotic regions provide distinct combinations of metabolic and inflammatory stress, but available evidence supports heterogeneous DC responses rather than a uniform compartment-specific state. Proposed relationships among local hypoxia, lipid exposure, pDC activity, and maturation-associated programs therefore remain mechanisms to be tested rather than fixed features of this anatomical compartment.

### 4.4 Adventitial ATLOs: local antigen presentation and candidate DC functions

The adventitia represents a spatially organized immune compartment in which advanced atherosclerosis may promote ATLOs containing B-cell follicles, T-cell zones, high endothelial venules, and antigen-presenting cells (116). Experimental studies show that these structures can recruit lymphocytes, support local antigen presentation, and organize adaptive immune responses outside the intimal plaque (33, 117, 118).

cDC2s are plausible participants in this environment because of their MHC-II-mediated antigen-presenting and CD4^+^ T-cell-instructing functions. However, the presence of an antigen-presenting compartment does not establish preferential cDC2 enrichment or cDC2 dominance in ATLO organization or T-cell priming, particularly in humans. Regulatory pathways also indicate that local immune responses within organized lymphoid structures need not be uniformly pathogenic (119, 120).

A working model proposes that ATLO-associated lymphocyte activation and DC trafficking connect adventitial niches, plaques, and draining lymphoid tissues (32, 33, 62). The direction and temporal sequence of this trafficking, and any dominant role for cDC2s, remain unproven. Human coronary-tissue studies support adventitial tertiary lymphoid structures, whereas spatial single-cell analyses have identified tertiary lymphoid organ-like structures within human plaques (31, 121). These observations concern related but anatomically distinct lymphoid environments and should not be treated as interchangeable evidence for a single DC circuit.

### 4.5 Spatial functional integration and technical limitations

Interpretation of plaque spatial data is limited by cellular annotation, spatial resolution, three-dimensional tissue structure, and disease heterogeneity.

DC–macrophage discrimination remains challenging, particularly in spatial transcriptomic datasets with multicellular spots, where abundant macrophage signals may obscure rare DC populations. Spatial resolution should therefore be complemented by lineage-informed annotation and independent phenotypic validation.

Cellular proximity identifies local neighborhoods but does not establish functional interaction. Likewise, inferred ligand–receptor pairs indicate candidate communication pathways rather than active signaling. Multiplexed imaging can strengthen anatomical evidence, whereas functional assays or targeted perturbation are required to establish specific mechanisms.

Two-dimensional tissue sections also incompletely capture the three-dimensional organization of plaques, limiting interpretation of apparent proximity among cells and anatomical compartments. Three-dimensional reconstruction can improve anatomical assessment but cannot resolve incorrect cellular annotation.

Most human plaque datasets are cross-sectional and vary by vascular bed, lesion stage, and clinical setting. Spatial associations therefore cannot establish whether a DC state precedes, contributes to, or follows local tissue injury. Plaque DCs should consequently be interpreted by integrating lineage, functional state, anatomical location, and evidence strength, with clear distinction among human observations, experimentally supported mechanisms, and proposed spatial relationships. **Table 4** summarizes the available evidence and principal validation gaps.

## 5 Therapeutic modulation of dendritic cell states in atherosclerosis: evidence and selectivity

DCs regulate atherosclerosis through antigen presentation, T-cell polarization, cytokine production, lipid handling, and peripheral tolerance (123). For therapeutic evaluation, interventions should be distinguished according to whether they are delivered directly to DCs, target DC-enriched states or pathways, or indirectly influence DC activity through pathways shared with other cells. Each strategy should be assessed in relation to its intended lineage or functional state, relevant anatomical niche and disease stage, supporting evidence, cellular selectivity, and potential effects on protective immune functions.

The therapeutic approaches discussed below are supported predominantly by mechanistic and preclinical evidence rather than established clinical efficacy. The evidence reviewed here does not establish sufficient cellular selectivity, efficacy, and long-term safety to support routine clinical use of these DC-associated approaches in atherosclerotic cardiovascular disease. The principal translational objective is therefore to modify disease-relevant DC states selectively while preserving antimicrobial immunity, antigen surveillance, and peripheral tolerance. **Figure 4** summarizes candidate intervention classes and the evidence required for their further development.

**Figure 4:**
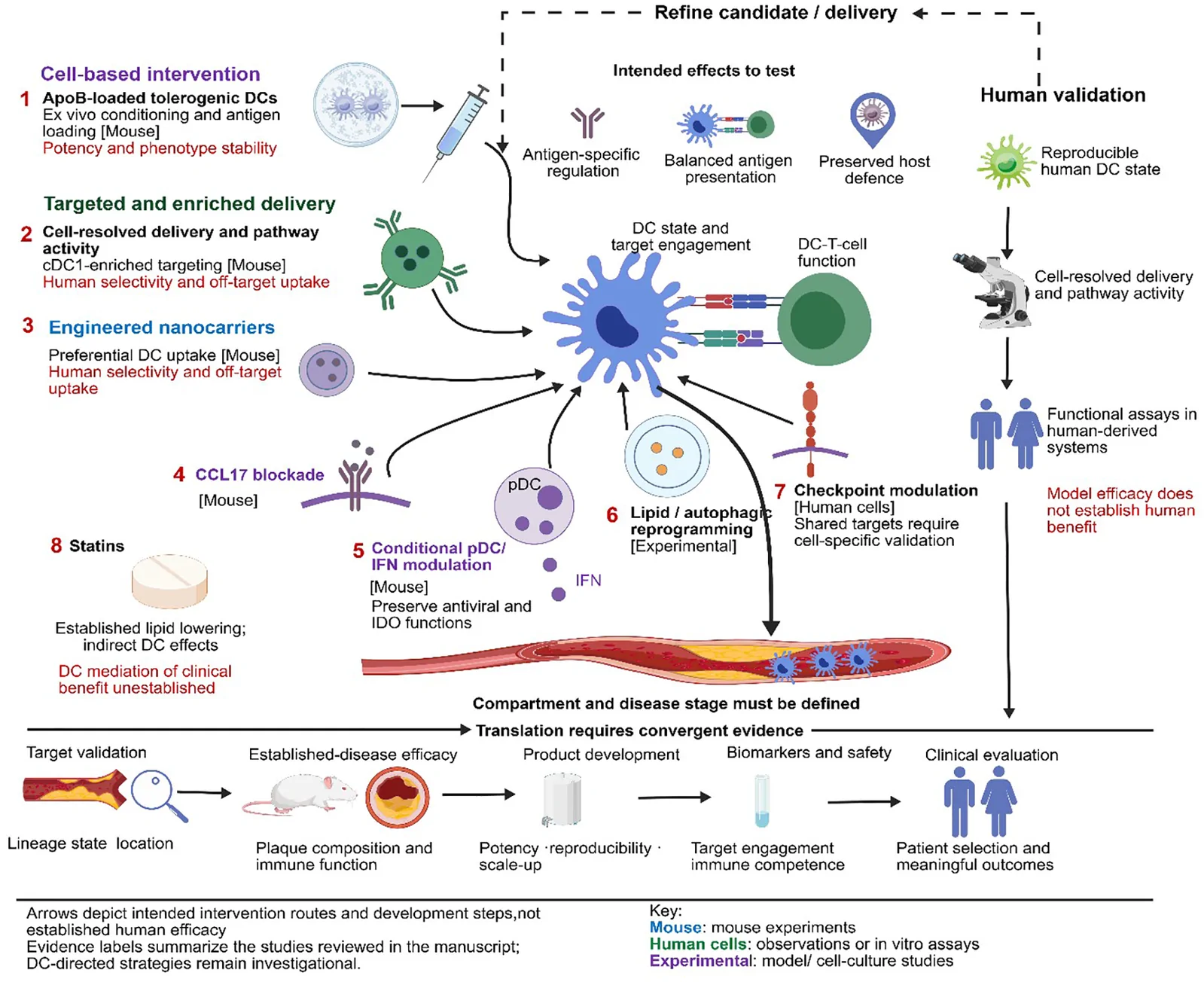
Translational roadmap for dendritic-cell-associated interventions in atherosclerosis. Candidate strategies include cell-based therapies, targeted delivery, pathway modulation, and indirect pharmacological approaches. Translation requires definition of the relevant DC lineage or state, anatomical context, target engagement, efficacy in established disease, and evaluation of product quality, safety, and immune competence. Human validation should confirm cellular selectivity and functional activity. Arrows indicate proposed intervention and development routes rather than established clinical efficacy.

### 5.1 Pathway-selective modulation rather than subset-specific therapy

Although current DC-directed approaches are sometimes described as subset specific, most candidate targets are neither restricted to a single DC lineage nor confined to the arterial wall. Single-cell studies show that inflammatory and metabolic programs are distributed across multiple immune and vascular populations (124, 125), and pathway expression may change after cells enter lesions. Current evidence therefore supports pathway-selective modulation more strongly than lineage-specific therapy. Such interventions should be distinguished from direct DC delivery and from systemic therapies that influence DC function only indirectly.

#### 5.1.1 CCL17-centered immune regulation

CCL17-producing DCs represent a comparatively well-characterized pathway in experimental atherosclerosis. Genetic Ccl17 deficiency and antibody-mediated CCL17 blockade increased Treg abundance and reduced lesion development in mice, supporting a causal role for a CCL17-associated pathway in restricting regulatory T-cell homeostasis (85, 86).

However, CCL17 is not exclusive to cDC2s and may be produced by multiple myeloid populations. CCL17 blockade should therefore be classified as a pathway-directed intervention rather than a cDC2-specific therapy. Before human translation, studies should identify the relevant cellular sources, spatial organization, responsive lymphocytes, arterial-wall pathway activity, and biomarkers of target engagement.

#### 5.1.2 Conditional modulation of pDC nucleic acid sensing

Plaque-derived nucleic acids may activate pDC-associated endosomal sensing and induce type I IFN production, providing a rationale for pathway inhibition (126, 127). However, experimental studies support both inflammatory and protective pDC functions: IFN-associated activity may amplify inflammation, whereas pDC depletion can remove IDO-associated regulatory activity (27).

Broad pDC depletion or sustained inhibition of nucleic-acid sensing could therefore remove protective functions and impair antiviral immunity. Therapeutic inhibition should be considered only where a pathogenic IFN-high pDC state is demonstrated using lesion-linked biomarkers rather than inferred from circulating IFN signatures alone. This strategy should therefore be regarded as state-directed in principle, but current systemic approaches do not selectively isolate pathogenic pDC states.

#### 5.1.3 Context-dependent modulation of lipid processing and autophagy

Lipid processing and autophagy regulate DC survival, antigen presentation, cytokine production, and T-cell interactions. Their effects are not uniform: lipid-loaded peripheral DC subsets can accumulate ROS and activation-associated molecules while showing impaired naïve T-cell priming, whereas other DC populations retain antigen processing and CD4^+^ T-cell priming under hypercholesterolemic conditions (65, 66). Surface maturation markers therefore do not by themselves define the relevant therapeutic state.

Experimental enhancement of lipid handling provides proof of principle. In ApoE-deficient mice, 2,3,5,4′-tetrahydroxystilbene-2-O-β-D-glucoside (TSG) increased DC lipophagy, reduced maturation-associated indices, shifted the Treg/Th17 balance toward regulation, and reduced lesion area (128). However, metabolic and autophagic pathways are shared with macrophages, lymphocytes, endothelial cells, and smooth muscle cells, so systemic treatment cannot be interpreted as DC-specific from lesion reduction alone.

Establishing a DC-mediated therapeutic mechanism will therefore require direct measurement of the relevant metabolic or autophagic defect, cell-specific target engagement, comparison with other plaque populations, and efficacy testing after lesions are established. It also remains necessary to identify which human plaque DC states contain reversible defects in lipid processing.

### 5.2 Reprogramming DC function under lipid and inflammatory stress

State reprogramming seeks to restore regulatory or functionally balanced DC activity without eliminating the cells. Modified lipoproteins, hypoxia, and inflammatory signals can alter DC maturation, adhesion, migration, antigen presentation, and T-cell communication (61, 63, 124). Because these inputs can exert divergent effects, candidate reprogramming strategies require direct functional testing in disease-relevant DC populations (129).

Studies of lipid-exposed DCs show that surface phenotype alone does not reliably predict function. Depending on the experimental system, modified lipoproteins can increase maturation-associated markers, whereas DCs under hypercholesterolemic conditions may retain antigen processing and T-cell priming (64, 66). Therapeutic reprogramming should therefore be defined by measured changes in antigen presentation, cytokine output, checkpoint activity, or T-cell function rather than by maturation-marker expression alone (130).

Costimulatory and coinhibitory signaling provides a potential means of modifying immune-cell interactions in atherosclerosis (131, 132). In addition to the PD-1–PD-L1 axis, TIM-3 has been proposed as a potentially atheroprotective immune checkpoint, although its cellular sources and functional effects extend beyond DCs (133). Circulating myeloid DCs from patients with coronary artery disease have been reported to express lower PD-L1 levels, whereas experimentally increasing PD-L1 activity reduced T-cell activation *in vitro* (88). These observations support checkpoint regulation as a candidate DC–T-cell interface but do not establish a DC-specific therapeutic mechanism or show that systemic checkpoint stimulation improves vascular outcomes. Such interventions would require substantially greater cellular, state, and tissue selectivity because the same pathways contribute to antimicrobial and antitumor immunity.

Existing drugs may also modify DC states indirectly. In human cell-culture systems, statins attenuated oxLDL-induced DC maturation and inflammatory T-cell responses while increasing IL-10, TGF- β, and regulatory T-cell-associated effects (103, 104). These observations support indirect modulation of experimentally differentiated DCs, but statins act through multiple hepatic, vascular, and immune pathways and should not be classified as DC-directed therapy. Their established cardiovascular benefit cannot be attributed to DC modulation without evidence of DC target engagement and causal mediation.

A central challenge is whether a reprogrammed state persists after treatment withdrawal or renewed exposure to the plaque environment (134). Because modified lipoproteins and inflammatory signals can alter DC maturation and function (63, 64), future studies should determine whether dysfunctional human plaque DCs can be durably reprogrammed, whether the induced state remains stable under renewed inflammatory stress, and whether persistent DC-dependent effects occur in established lesions. This requirement is also relevant to tolerogenic DC vaccination, for which administered cells must retain their intended function in an inflammatory host environment.

### 5.3 Antigen-specific tolerogenic DC vaccination and related strategies

Antigen-specific approaches aim to modify disease-relevant immunity without broadly suppressing vascular inflammation (135–137). However, three intervention types should be distinguished: viable tolerogenic DC products generated or conditioned ex vivo and loaded with plaque-associated antigens; apoptotic DC interventions that act through uptake and secondary regulatory responses; and antigen-delivery platforms such as peptide-loaded nanoparticles that do not administer DCs as the therapeutic product. These approaches differ in mechanism, selectivity, manufacturing requirements, and evidence for direct DC involvement.

Preclinical studies support biological feasibility. Interleukin-10-treated DCs loaded with apolipoprotein B100 reduced aortic atherosclerosis and induced regulatory immune responses in hypercholesterolemic mice (138). In another model, vaccination with oxLDL-pulsed DCs before lesion induction reduced carotid atherosclerosis and improved plaque-stability measures (139). Transfer of oxLDL-induced apoptotic DCs was associated with increased CD103^+^ DCs and regulatory T cells, reduced lesion formation, and increased plaque collagen (81); these apoptotic preparations should be distinguished from viable tolerogenically conditioned DC products. ApoB100 peptide immunization delivered by nanoparticles also reduced experimental atherosclerosis (140), but represents an antigen-delivery platform rather than an ex vivo DC product.

These approaches should not be regarded as a single therapeutic class. Some involve ex vivo manipulated DCs, whereas others use antigen-delivery platforms in which DCs are secondary targets or intermediaries (138, 140–142). Across studies, antigen composition, conditioning, maturation or viability, route of administration, and treatment timing vary substantially (81, 138, 139). Moreover, several interventions were tested before or near lesion induction, so efficacy in early murine disease does not establish benefit in established human atherosclerosis.

Antigen selection and phenotypic stability present additional challenges. ApoB-derived and oxidation-associated antigens have shown activity in experimental models (138–140), but the relevant antigenic repertoire may vary across patients and disease stages. Because modified lipoproteins and inflammatory signals can alter DC maturation and function, tolerogenic products must retain their regulatory phenotype after administration (63, 64, 134).

Translation will therefore require reproducible product identity and manufacturing, functional potency criteria, stability under inflammatory conditions, efficacy after lesion establishment, and preservation of protective immunity. The studies reviewed here remain preclinical, and no study cited in this review establishes cardiovascular-event reduction with an atherosclerosis-specific tolerogenic DC product in humans. These limitations also motivate *in vivo* delivery strategies that avoid individualized ex vivo cell production.

### 5.4 Targeted delivery and pharmacological reprogramming

Targeted delivery may improve the anatomical and cellular selectivity of DC modulation, but it cannot compensate for an inadequately validated biological target. Accumulation of a carrier within a lesion or DC population does not demonstrate modification of the disease-relevant cell state. Direct DC delivery, DC-enriched delivery, and pathogenic-state-specific delivery should therefore be distinguished when evaluating therapeutic selectivity.

Nanoparticle structure, including morphology and ligand topology, influences biodistribution and immune-cell interactions (143–145). In mice, polymersome morphology increased delivery to splenic and plaque-associated DC-enriched populations relative to other nanoparticle configurations (146, 147). However, uptake by the mononuclear phagocyte system and limited discrimination between DCs and macrophages remain important constraints. Plaque accumulation alone does not demonstrate recognition of a pathogenic DC state; payload-matched controls and cell-resolved uptake measurements are therefore required.

Receptor-directed delivery may provide greater cellular enrichment. In a mouse study, anti-CLEC9A-decorated dexamethasone nanoparticles targeted cDC1s, reduced inflammatory T-cell responses, and decreased lesion formation without detectable impairment of the antiviral responses measured under the experimental conditions (74). However, the approach targeted cDC1s broadly rather than selectively recognizing pathogenic plaque cDC1 states. It therefore provides evidence of receptor-directed cellular enrichment, not pathogenic-state-specific therapy. Human translation requires confirmation of CLEC9A expression and accessibility, cell-resolved biodistribution, payload activity, efficacy in established lesions, and long-term immune safety.

Pharmacological agents should be classified according to their demonstrated selectivity. Statins and other systemic agents can alter DC differentiation, maturation, or vascular trafficking; for example, atorvastatin suppressed oxLDL-induced DC-like differentiation *in vitro* (148), and 3-hydroxy-3-methylglutaryl coenzyme A reductase inhibition reduced DC–endothelial interactions in an experimental system (61). Their established clinical effects cannot be attributed to DC modulation without evidence of DC target engagement and causal mediation. These agents may serve as mechanistic probes or nanoparticle payloads but are not currently DC-specific therapies.

Preclinical studies should compare targeted and untargeted formulations with identical payloads, quantify uptake across major plaque cell populations, and determine whether efficacy depends on the intended DC population or pathway. Relevant outcomes should extend beyond lesion area to plaque composition, necrotic-core size, fibrous-cap integrity, immune competence, biodistribution, toxicity, and durability. These are proposed criteria for evaluating selectivity and mechanism rather than conclusions established by individual nanoparticle studies.

Targeted delivery can facilitate DC modulation but does not by itself establish therapeutic precision. Translation will require confirmation of selectivity in human tissues, direct target engagement in the intended DC state, efficacy in established lesions, reproducible manufacturing, controlled systemic biodistribution, and long-term immune safety.

### 5.5 Current evidence boundary and translational position

DC-associated interventions remain investigational. Current evidence derives mainly from mechanistic and efficacy studies in mice, whereas human evidence is largely limited to cellular phenotyping, plaque profiling, circulating-cell observations, and *in vitro* experiments (30, 108). Because many candidate targets are shared with macrophages, lymphocytes, and vascular cells, reduced lesion area after systemic treatment does not establish a DC-specific mechanism without selective target engagement and causal dependence on the intended population.

For clinical translation, future studies should validate the relevant human DC state, define the anatomical compartment and disease stage in which it is therapeutically relevant, demonstrate preferential delivery or pathway engagement, test the intended functional mechanism, evaluate efficacy in established disease, develop pathway-specific biomarkers, and assess preservation of antimicrobial immunity, tumor surveillance, and peripheral tolerance. The evidence reviewed here does not establish that any current strategy satisfies all of these requirements. **Table 5** summarizes the intended DC population or state, anatomical context or disease stage, supporting evidence, demonstrated selectivity, and principal translational requirement of each major strategy.

**Table 5:** Targets, disease context, evidence, and translational requirements of candidate DC-modulating strategies.

Strategy and intervention type	Intended DC population or state	Anatomical context or disease stage	Supporting evidence	Demonstrated selectivity and its limits	Principal translational requirement
CCL17-pathway blockade; pathway-directed intervention	CCL17-associated DC–T-cell circuit restricting Treg homeostasis; not a cDC2-exclusive target	Plaque and lymphoid immune responses; antibody blockade tested during murine disease progression. The treatment-responsive human compartment and stage remain undefined.	*Ccl17* deficiency and antibody blockade increased Tregs and reduced murine atherosclerosis (85); subsequent work further defined the associated receptor pathway (86).	Targets a chemokine pathway rather than selectively delivering treatment to a DC lineage.	Identify relevant human source cells, pathway activity, response biomarkers, and effects on protective immunity.
Conditional pDC/IFN modulation; proposed state-directed intervention	Pathogenic IFN-producing pDC activity, while preserving IDO-associated regulation	Human plaque pDC observations and experimental aortic pDC studies; no validated compartment- or stage-selective treatment approach.	Human plaque experiments support IFN-associated inflammatory effects (93), whereas selective pDC depletion aggravated disease in a model with protective IDO-associated regulation (27).	Systemic nucleic-acid-sensing or IFN inhibition does not selectively isolate pathogenic pDC states.	Establish lesion-linked biomarkers and demonstrate benefit without disrupting antiviral or regulatory functions.
DC metabolic-state reprogramming; shared-pathway intervention	Lipid-handling, lysosomal, and autophagic programs affecting DC function; no established lineage-specific target	Lipid-exposed experimental DCs and diet-induced murine lesions; reversible defects in specific human plaque DC states remain unresolved.	TSG treatment was associated with DC lipophagy-related changes, altered Treg/Th17 balance, and reduced murine plaque area (128). Other experiments show that lipid loading can preserve antigen presentation (66).	Systemic metabolic modulation is not DC-specific; lesion reduction alone does not establish DC-dependent efficacy.	Demonstrate autophagic flux, DC target engagement and causal contribution, and efficacy in established disease.
Checkpoint-interface modulation; shared-pathway intervention	PD-1/PD-L1-associated DC–T-cell regulation; TIM-3 as a related proposed target	Human circulating myeloid DCs and *in vitro* interactions; therapeutic targeting of a defined plaque compartment or stage is not established.	Human CAD samples showed reduced myeloid-DC PD-L1 expression, and increased PD-L1 expression *in vitro* reduced T-cell stimulation (88). TIM-3 represents a related candidate pathway rather than equivalent DC-specific therapeutic evidence (133).	These pathways operate across multiple cell populations; the human DC experiments do not establish selective vascular treatment.	Identify the relevant cellular source and therapeutic window while preserving antimicrobial and antitumor immunity.
Antigen-specific tolerogenic DC product; ex vivo cell-based intervention	Antigen-loaded, tolerogenically conditioned DCs intended to induce regulatory responses	Systemically administered cells in hypercholesterolemic mice; no validated human plaque-compartment targeting or treatment window.	IL-10-conditioned, ApoB100-loaded DCs reduced aortic lesions and induced regulatory immune responses in mice (138).	The administered product consists of manipulated DCs, but this does not establish plaque-restricted delivery or equivalent human antigen specificity.	Establish product identity, functional potency, antigen selection, manufacturing consistency, and stability under inflammatory conditions.
DC-enriched nanocarrier delivery; delivery-enriched intervention	DC populations preferentially taking up engineered carriers; not necessarily a single lineage or pathogenic state	Splenic and plaque-associated DC populations in mice; human distribution and stage-dependent accessibility require validation.	Polymersome morphology and surface engineering enhanced DC delivery (146). An antigen/immunomodulator-loaded formulation reduced experimental lesion development (147).	Relative enrichment was demonstrated in the tested systems, but neither exclusive DC uptake nor selective recognition of a disease-causing state should be assumed.	Confirm cell-resolved biodistribution, payload-matched efficacy, DC-dependent action, and repeated-dose safety.
CLEC9A-directed nanotherapy; receptor-directed delivery	CLEC9A-associated cDC1 delivery of dexamethasone to reduce inflammatory immune activity	Systemic administration in atherosclerotic mice; not restricted to plaque-resident pathogenic cDC1s.	CLEC9A-targeted nanoparticles reduced lesions and inflammatory T-cell responses; measured antiviral responses were not detectably impaired under the tested conditions (74).	cDC1-directed delivery was demonstrated in the mouse model; pathogenic-state and plaque-specific selectivity were not established.	Validate human receptor accessibility, cellular targeting, established-disease efficacy, and broader long-term immune safety.
Statins as indirect DC-modulating agents; systemic pharmacological intervention	HMG-CoA reductase-related pathways with secondary effects on DC maturation and immune interactions	Human cell-culture and experimental systems; clinical lipid-lowering use does not identify a DC-specific treatment niche.	Statins altered oxLDL-conditioned DC–T-cell responses in human experimental systems (103, 104).	No DC-specific delivery; clinical cardiovascular benefit cannot be assigned to DC modulation from these experiments alone.	Establish DC target engagement and causal mediation before interpreting clinical benefit as DC-directed efficacy.

## 6 Translational barriers and future directions

Although experimental findings support continued investigation, translation remains limited by uncertainty regarding whether DC populations and functional states identified in experimental models have equivalent counterparts in human plaques, remain accessible at clinically relevant disease stages, and can be modified without disrupting protective immunity.

### 6.1 Where the current evidence is biased

Mouse models dominate causal evidence because conditional deletion, temporally controlled depletion, and lineage tracing can distinguish developmental effects from functions acquired during disease. Human studies rarely provide comparable experimental control. Standard murine models reproduce lipid accumulation and chronic arterial inflammation but do not reproduce every feature or the full natural history of clinically unstable human atherosclerosis; conclusions regarding plaque rupture and clinical events should therefore remain model specific.

Plaque instability illustrates this limitation. Rupture occurs infrequently in conventional atherosclerotic mice despite being a major cause of acute coronary and cerebrovascular events in humans. Accelerated or locally modified vascular models can generate features associated with lesion instability and are useful for testing defined mechanisms (149). However, altered hemodynamics, vascular injury, or procedure-associated inflammation may themselves modify DC recruitment and activation. The resulting DC states may therefore reflect both the developing lesion and the experimentally imposed conditions.

Disease-stage mismatch is equally important. Many interventions are tested during lesion initiation or progression and primarily report lesion burden (150, 151), whereas advanced human plaques develop over years of cell death, repair, and extracellular-matrix remodeling and may contain large necrotic cores, thin or disrupted fibrous caps, calcification, and intraplaque hemorrhage. Immune composition and cellular state can also vary with vascular bed and lesion stage (30, 108).

Accordingly, reduced lesion area in an early-disease model does not establish efficacy against an established unstable plaque. A treatment may delay lipid accumulation or leukocyte recruitment without stabilizing an existing fibrous cap or reducing necrosis. Studies intended to support treatment of established disease should therefore initiate intervention after lesion formation and evaluate fibrous-cap structure, necrotic-core area, vascular inflammation, and model-appropriate complications separately from lesion burden (149).

### 6.2 Uneven cross-species conservation across DC populations

Murine and human DC populations cannot be assumed to be directly equivalent, and the degree of conservation differs among lineages and functional states. cDC1s provide a comparatively well-defined correspondence, with conserved IRF8–BATF3-associated developmental features (30, 71). However, conserved developmental identity does not establish equivalent behavior in atherosclerotic tissue, where lipid loading, cytokines, and local stress may modify function differently across species.

The cDC2 compartment is more difficult to align because human CD1c-associated populations are heterogeneous and do not map uniformly onto murine functional states. Cross-species interpretation of pDCs is similarly complex: TCF4-associated developmental programs and commonly used human markers such as CD123 and BDCA-2 do not provide a one-to-one functional correspondence (152). A murine cDC2 or pDC state should therefore not be assigned to a human plaque population solely on the basis of a limited set of shared markers.

These uncertainties have direct therapeutic implications. Computational integration can identify candidate counterparts, but cannot establish equivalent developmental origin, target accessibility, function, or response to treatment. Receptor-directed delivery requires confirmation that the intended human population expresses an accessible target, whereas pathway-directed interventions additionally require assessment of the same pathway in macrophages, lymphocytes, and vascular cells.

Human blood and lymphoid populations can provide useful reference states before corresponding populations are examined in arterial tissue (153), but they cannot substitute for plaque-specific validation. Shared myeloid markers, rare-cell recovery, and tissue-processing procedures can distort annotation, and these errors may propagate into downstream spatial and functional analyses (71, 83, 84). Functional studies should therefore specify whether the tested cells are circulating DCs, plaque-derived DCs, or experimentally differentiated cells rather than treating these preparations as equivalent.

### 6.3 Therapeutic window, patient selection, and biomarkers

Conservation of a pathway does not establish its suitability as a therapeutic target. Treatment timing is particularly important because many animal studies initiate intervention before substantial lesion formation or shortly after atherogenic feeding begins, whereas clinical treatment would usually occur after disease is established and alongside lipid-lowering, antiplatelet, and other secondary-prevention therapies. Preventing further inflammatory destabilization and reversing an extensively calcified or fibrotic lesion are distinct therapeutic objectives.

Patients with residual inflammatory activity after an acute coronary syndrome may represent a candidate population for selected mechanism-based interventions. However, this is not an established indication for DC-directed therapy, and acute ischemic injury itself alters myelopoiesis and immune-cell trafficking. Biomarkers measured shortly after an event may therefore reflect tissue injury or systemic immune responses rather than activity of the plaque pathway intended for treatment. Sampling schedules and concomitant therapy should be considered when interpreting target activity.

Primary prevention presents a different risk–benefit profile because systemic immune modulation must be weighed against lower baseline event risk and possible adverse effects (154). Off-target effects and disruption of protective DC functions are particularly relevant because DC antigen presentation contributes to immune responses against infectious stimuli (155). At the other extreme, very advanced disease may be less responsive to modulation of a single immune pathway because extensive structural injury has already occurred. Therapeutic objectives should therefore remain stage appropriate rather than assume one window for all patients.

Disease stage alone is also insufficient for patient selection. Patients with similar plaque burden or stenosis may differ in morphology and inflammatory activity (156). Biomarkers used in a mechanism-based trial should measure the pathway being targeted rather than inflammation in general. C-reactive protein may identify residual inflammatory risk, but cannot establish activity of a CCL17-associated, pDC-interferon, or tolerogenic DC program. Without a pathway-linked marker of target engagement, a negative result may reflect an invalid mechanism, inadequate exposure, or biologically unsuitable patient selection.

### 6.4 From cross-species alignment to human functional validation

Single-cell integration and spatial methods can identify candidate human DC populations, transcriptional programs, and tissue neighborhoods (30, 35, 157). These approaches provide important context but do not establish developmental equivalence or functional causality. Reliable cellular annotation should therefore precede mechanistic interpretation, particularly because misclassification of macrophages or monocyte-derived cells can propagate through spatial mapping, trajectory analysis, and ligand–receptor inference.

Human sampling introduces additional limitations. Most arterial specimens are obtained at surgical end points from patients with advanced disease, vascular beds differ in hemodynamics and lesion composition, medication histories vary, and tissue processing can alter cell recovery. Fluorescence-activated sorting itself has been associated with depletion of particular plaque populations and inflammatory transcriptional changes in recovered cells (84). Technical bias and biological heterogeneity should therefore be evaluated separately rather than collapsed into a generic batch effect.

Human-derived DCs, plaque cells, organotypic cultures, and plaque explants provide complementary systems for functional testing. Experiments should specify cellular provenance, directly perturb the proposed pathway, and measure the predicted immune or vascular response rather than target expression alone. Where a DC-mediated effect is claimed, direct effects on responding T cells or vascular cells should be excluded. For tolerogenic interventions, reduced T-cell stimulation alone is insufficient: evidence should demonstrate a relevant regulatory function, such as antigen-specific suppression or induction of functionally suppressive Tregs, while excluding cell injury, reduced viability, or loss of antigen-processing capacity as alternative explanations.

A credible translational pathway should connect stage-appropriate efficacy, a validated human cellular target, relevant tissue localization, directly tested function, pathway-linked biomarkers, and acceptable immune safety. Longitudinal evidence would strengthen this chain, although repeated plaque sampling is rarely feasible. Serial blood measurements and vascular imaging may complement carefully annotated tissue end points when their relationship to the proposed plaque mechanism is demonstrated rather than assumed. **Table 6** summarizes the minimum evidence needed to support further development.

**Table 6:** Proposed translational validation framework for DC-associated interventions in atherosclerosis.

Validation domain	Evidence supporting progression	Implication for further development
Disease stage and therapeutic objective	Benefit after lesion establishment when established disease is the intended indication; separate assessment of lesion burden, cap structure, necrosis, inflammation, and model-appropriate complications.	Early-lesion efficacy supports further testing, not claims of advanced plaque stabilization or prevention of clinical events.
Human cellular target and tissue compartment	Independent confirmation of the intended human population, target expression, and localization in the relevant vascular compartment, accounting for disease stage and vascular bed.	Reassess targeting if the target is absent, inaccessible, or predominantly expressed by unintended cells.
Human functional response	Controlled target perturbation in clearly characterized human-derived cells or tissue systems, with reproducible effects on the proposed immune or vascular response.	Distinguish DC-mediated effects from effects on other cells. For tolerogenic strategies, demonstrate regulatory function rather than reduced stimulation caused by cell injury, reduced viability, or cellular dysfunction.
Biomarkers and target engagement	A measurable indicator linked to the intended mechanism, supported by exposure–response data and, where feasible, tissue validation.	Distinguish inadequate exposure or engagement from failure of the proposed mechanism; general inflammatory markers do not establish DC-specific activity.
Therapeutic window and patient selection	Evidence that the target remains present and modifiable during the proposed treatment window, with analyses accounting for acute events and concomitant therapies.	Define a biologically plausible treatment population without assuming that a candidate biomarker is already validated for patient selection.
Selectivity, delivery, and safety	Characterization of cellular uptake, biodistribution, dose–response relationships, and effects on protective antigen presentation and immune regulation.	Optimize delivery or reconsider development when the desired response cannot be separated from unacceptable immune disruption.
Integrated development decision	Convergence of stage-relevant efficacy, human target validation, functional response, feasible exposure, and a defensible safety profile.	Advance only when the relevant prerequisites are met; otherwise identify the missing experiment, revise the strategy, or discontinue development.

## 7 Conclusions and future perspectives

Current evidence supports DCs as important regulators of vascular immunity in atherosclerosis, but does not support assigning a fixed pathogenic or protective function to an entire DC lineage (158, 159). Developmental identity defines functional potential, whereas activity in diseased arteries is modified by antigen exposure, inflammatory and metabolic signals, neighboring cells, anatomical location, and disease stage. Experimental manipulation provides causal evidence in defined models, but differing outcomes across systems emphasize that lineage identity and functional effect are not equivalent.

Human single-cell, multimodal, and spatial studies have substantially improved resolution of plaque-associated DC populations and maturation programs. However, transcriptional annotation, ligand–receptor inference, and spatial proximity do not by themselves establish developmental origin, antigen presentation, immune regulation, or causal effects on plaque stability. Candidate human DC states therefore require convergent identification and direct functional testing. Reduced T-cell stimulation should not be interpreted as tolerance unless accompanied by regulatory activity and exclusion of cellular dysfunction or loss of viability.

The translational priority is consequently not to identify an increasing number of DC subsets, but to connect reproducible human cellular identity with disease-relevant functional state, anatomical context, and experimentally demonstrated mechanism. Candidate interventions should distinguish direct DC targeting from pathway-directed or indirect modulation, demonstrate target engagement in the intended population, and be tested after lesion establishment when established disease is the therapeutic objective. Pathway-linked biomarkers and preservation of antimicrobial immunity, tumor surveillance, and peripheral tolerance will also be required. Until these conditions are met, DC-associated therapeutic strategies should remain investigational rather than clinically validated approaches to atherosclerotic cardiovascular disease.
